# A CLSTM and transfer learning based CFDAMA strategy in satellite communication networks

**DOI:** 10.1371/journal.pone.0248271

**Published:** 2021-03-10

**Authors:** Qiang He, Zheng Xiang, Peng Ren

**Affiliations:** School of Telecommunications Engineering, Xidian University, Xi’an, China; Fuzhou University, CHINA

## Abstract

With the development of the economy and technology, people’s requirement for communication is also increasing. Satellite communication networks have been paid more and more attention because of their broadband service capability and wide coverage. In this paper, we investigate the scheme of convolutional long short term memory (CLSTM) network and transfer learning (TL) based combined free/demand assignment multiple access (CFDAMA) scheme (CFDAMA-CLSTMTL), which is a new multiple access scheme in the satellite communication networks. Generally, there is a delay time T between sending a request from the user to the satellite and receiving a reply from the satellite. So far, the traditional multiple access schemes have not processed the data generated in this period. So, in order to transmit the data in time, we propose a new prediction method CLSTMTL, which can be used to predict the data generated in this period. We introduce the prediction method into the CFDAMA scheme so that it can reduce data accumulation by the way of sending the slots request which is the sum of slots requested by the user and the predicted slots generated in the delay time. A comparison with CFDAMA-PA and CFDAMA-PB is provided through simulation results, which gives the effect of the CFDAMA-CLSTMTL in a satellite communication network.

## 1. Introduction

In recent years, wired and wireless communication technologies have been greatly developed. As we all know, terrestrial communication networks have been able to meet the needs of high-speed and wide bandwidth communication for users in not only urban but also countryside at a very low cost. However, it is not possible to achieve seamless global interconnection, especially for those people in some remote areas. The main reason why the service could not be established for these areas is that we can not deploy terrestrial communication system conveniently and economically. With this regard, satellite communication networks are regarded as an indispensable and complementary communication method in future communication systems [1].

In recent years, satellite communication networks have been paid more and more attention. In research and industry, people pay attention to how to promote the ability to provide broadband radio access and so on. Because the satellite communication networks can provide high performance of QoS at a very low cost, therefore, it has to be applied more and more widely no matter in the field of fixed or mobile communication systems. In the coming 5G or even 6G era, the satellite communication network is regarded as the most important part of the integrated network [2–5]. The practice has shown that widely deployed integrated terrestrial-satellite networks, along with emerging techniques, such as MIMO (Multiple-input Multiple-Output) [6], OFDM (orthogonal frequency division multiplexing) [7], and cognitive radio (CR) [8], can promote the performance of communication and provide satisfactory services for the growing number of terminals within limited resources.

It is noteworthy that satellite bandwidth is a limited and expensive commodity, which is the key point that limits its widespread and long-term usage. It must be used as efficiently as possible as it is such a scarce resource. So far, the main access protocols of satellite communication networks are OMAs (orthogonal multiple access schemes) [9], which are shown in Table 1. As shown in this table, it can be divided into three categories, in which frequency-division multiple access (FDMA), time-division multiple access (TDMA), and code-division multiple access (CDMA) are fixed assignment protocols, while ALOHA and demand assignment multiple access (DAMA) schemes are random assignment and a demand assignment, respectively. The application of FDMA/TDMA/CDMA schemes can provide access services when the number of users is small and can effectively avoid multiple access interference. For the OMAs, the orthogonality between users limits the number of users in the system as well as further improvement on the spectrum efficiency. Obviously, if we use the ALOHA protocol in a network with high number of terminals, there will be a lot of conflicts in the network. Moreover, the use of DAMA requires network resource occupation. As the multiple access protocols are not suitable for future networks, there is an urgent need for new multiple access schemes, which can harmoniously integrate with OMA techniques. Therefore, the motivation and purpose of this paper is to design a new multiple access control (MAC) protocol for the existing satellite communication networks.

**Table 1:** Typical multiple access model.

Category	Scheme	Key characteristics	Drawback
Fixed assignment	FDMA	Users send messages in different frequency/time/code domains	● Inefficient resource utilization ● Low system capacity
	TDMA		
	CDMA		
Random assignment	ALOHA	Users send data directly when they need to	● Data collision prone ● No guarantee in QoS
Demand assignment	DAMA	Dynamic allocation of resources according to user’s requests	● Additional allocation of scheduled resources is required ● Time slots need to be captured for an implicit reservation

Recently, there is a novel multiple access protocols, named combined free demand assignment multiple access, which is abbreviated as CFDAMA in this paper [10]. The CFDAMA scheme is a TDMA based access scheme, which is proposed by Mohammed et al [11]. It has been designed to provide significant improvements in the delay/utilization performance of satellite channels supporting a finite number of users with burst data traffic. CFDAMA is a combination of free assignment of time slots and demand assignment. With this new scheme, the satellite networks can provide a minimum end-to-end delay.

With the rapid growth of the number of users and their data traffic [12], the wireless network will face much more severe challenges. For the satellite communication networks, it is very important that the multiple access protocol must provide convenient access capabilities no matter whenever and wherever. However, the current CFDAMA scheme and its modified scheme are difficult to meet the demands. For example, it is proved that the CFDAMA scheme can get a good performance in the case of a low burst source [13]. However, the performance will be greatly affected when the source model is ON-OFF, the channel load is large, and the number of user is large.

In this paper, we proposed a CFDAMA scheme based on CLSTM and transfer learning (CFDAMA-CLSTMTL) in the satellite communication system. The motivation is to improve the performance of the multiple access scheme in the satellite communication system by the CLSTM and transfer learning combined with CFDAMA, which is used to predict the data traffic in the network. The contributions of this paper are summarized as follows:

1. We propose a prediction-based strategy to solve the problem of data accumulation in the period of time T, which is also called the satellite onboard processing time. According to the traditional CFDAMA protocol, the users apply for resource from the satellite in the light of the data in the send queue, while not consider of the data generated in the satellite onboard processing time. From that point, the traditional multiple access schemes will increase the data accumulation. So, to transmit the data in time, we propose a new prediction strategy.
2. We propose a new prediction strategy which combines CLSTM with transfer learning. This new strategy makes full use of the advantages of CLSTM and transfer learning, which can improve the performance of the prediction for the CLSTM networks when the period of time increases (the prediction efficiency of the CLSTM networks become worse when the period of time increases, and the transferring learning can solve these problems effectively).

The rest of this paper is organized as follows. At the beginning of Section II, we will present some preliminary studies and then introduce related research. In Section III, we will introduce some basic concepts of CLSTM and transfer learning, and propose the CFDAMA-CLSTMTL scheme, and then introduce it in detail. In Section IV, we will present the experimental results and analyze the results. In Section V, we will draw a summary of this paper and outline the future research directions.

## 2. Research on the CFDAMA scheme

There are many MAC protocols proposed in satellite communication networks, and CFDAMA is one of them which has been widely used. The principle of the CFDAMA scheme is to assign the slots in a frame to the user on-demand firstly, and then assign the remaining slots in the system one by one in a freeway, so as to ensure the rationality and fairness for resource assignment of the satellite. The CFDAMA scheme combines the advantages of on-demand assignment and free assignment and can provide a better performance in the satellite communication networks.

### 2.1. Principle of the CFDAMA scheme

A scheduler is important for a communication system. It is assumed that there is a satellite onboard scheduler, by which the user can access the communication system in a short DAMA request to acknowledgment time. The CFDAMA scheduler maintains a reservation request table and a free assignment table [10,11,14]. The reservation request table (shown in Table 2) queues requests for demand assigned slots. Once a request is received by the scheduler, it will place an entry on the bottom of the reservation request table indicating the identity (ID) of the requesting user and the corresponding number of slots requested. The free assignment table (shown in Table 3) consists of the ID numbers of all active terminals in the system.

**Table 2:** Reservation request table.

ID of user 0	Number of slots requested by user 0	Number of slots assigned to user 0
ID of user 1	Number of slots requested by user 1	Number of slots assigned to user 1
ID of user 2	Number of slots requested by user 2	Number of slots assigned to user 2
-------------	-------------	-------------
ID of user N	Number of slots requested by user N	Number of slots assigned to user N

**Table 3:** Free assignment table.

ID of user 0	Number of free slots assigned to user 0
ID of user 1	Number of free slots assigned to user 1
ID of user 2	Number of free slots assigned to user 2
-------------	-------------
ID of user M	Number of free slots assigned to user N

The satellite on-board scheduler assigns slots on a frame-by-frame basis, transmitting the information on a time division multiplex (TDM) downlink [15]. In the first instance, the scheduler will serve entries from the top of the reservation request table by demand assigning contiguous slots to the corresponding user, based on the number of slots requested. In the absence of any queued requests, the scheduler will freely assign slots to users in a round-robin fashion. This is achieved by assigning successive slots, one by one, to the user currently at the head of the free assignment table, moving each user to the bottom of the table after each slot allocation. To provide users that have not received a slot for a long time a better chance of obtaining a free assigned slot, each time a user receives demand assigned slots and is removed from the reservation request table, it is also moved to the bottom of the free assignment table.

**Fig 1:**
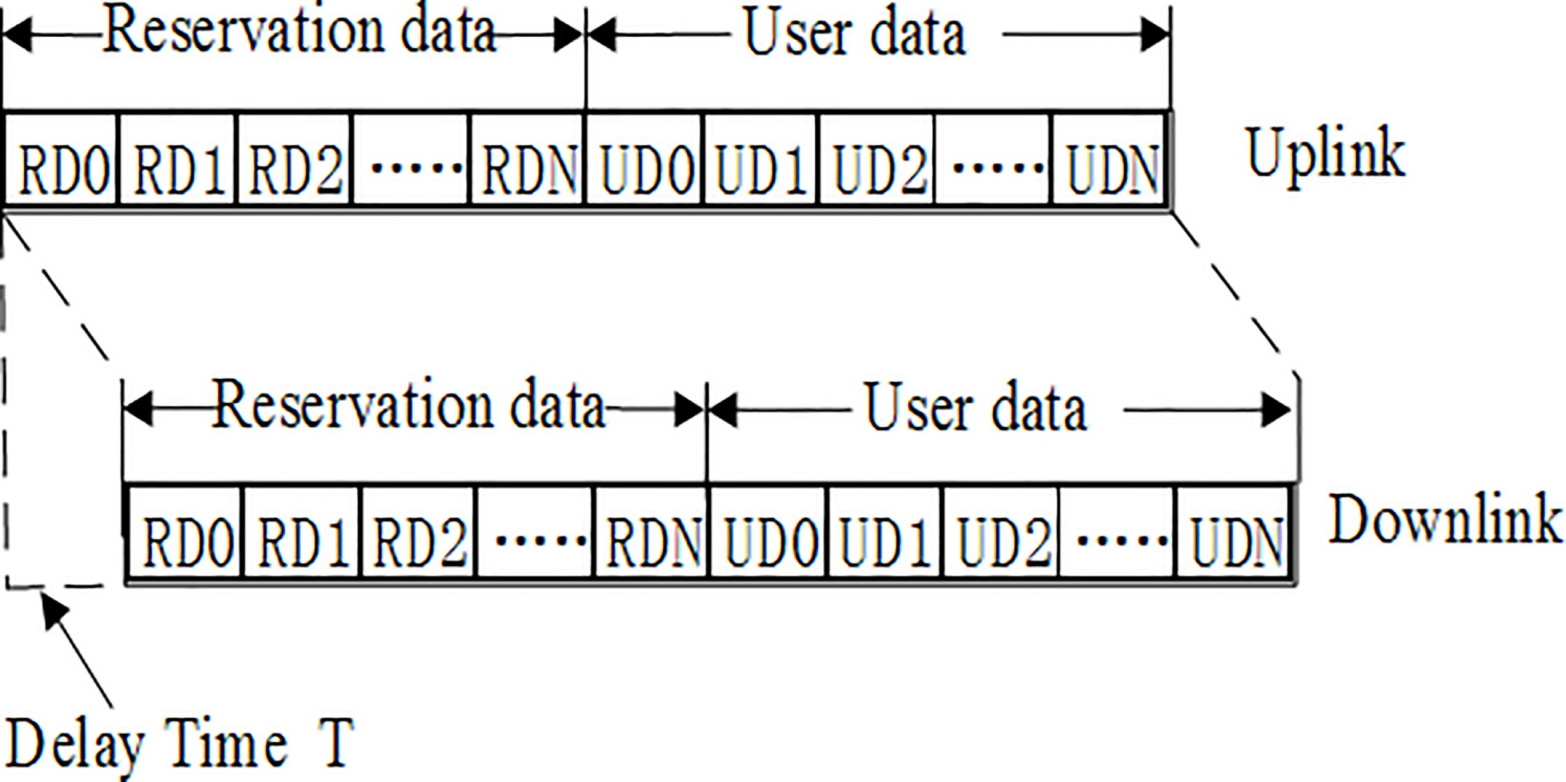
Uplink/downlink frame format of CFDAMA.

The uplink/downlink frame format of the CFDAMA scheme [16] is shown in Fig 1, where RD represents the reservation data, and UD represents the user data. The uplink frame includes reservation data and user data. The reservation data is used for loading the reservation requests issued by users, while the user data is used for loading the data sent by users. A satellite onboard scheduler assigns the number of slots to users on-demand or freely, and then users send data in the corresponding slots assigned to them.

The downlink frame also includes reservation data and user data, where the reservation data is used for loading the response information assigned by the satellite onboard scheduler to all the reservation requests. This information includes the user’s data transmission slots and the free assignment information assigned by the satellite. User data is also used for loading the data sent by users. There is a time delay between the uplink and downlink frames, which represents the satellite on-board processing time.

### 2.2. Related research

Many improved CFDAMA schemes have been proposed in these years [17]. There are two typical modified CFDAMA strategies given below. They are CFDAMA-PA (Pre-Assigned Request), and CFDAMA-PB (Piggy-Backing Request). A more detailed description of the mentioned two strategies is described as follows.

#### 1. CFDAMA-PA

The CFDAMA-PA [18] scheme, which is also known as CFDAMA-Pre Assigned, is the CFDAMA scheme based on scheduled slots. The uplink/downlink frame format of CFDAMA-PA is just the same as CFDAMA. In the CFDAMA-PA scheme, all users have their own fixed request slots in the uplink frame, and all users send their requests in its fixed slots.

Each user will make a request if required. The number of slots requested is given by formula (1).

NSR=NPQ–NOR (1)

NSR = Number of slots requested

NPQ = Number of packets queued

NOR = Number of outstanding requests

#### 2. CFDAMA-PB

The CFDAMA-PB [19] scheme, which is also known as CFDAMA-Piggy Backing, is the CFDAMA scheme based on piggyback reservation. The uplink frame format is different from that of other strategies. In the CFDAMA-PB scheme, request slots are embedded with the uplink data transmission slots. The users make requests accompanying their data packets transmission. If required, the user will make a request accompanying its data packets transmission. The number of slots required by users is given by formula (3).

NSR=(NPQ–1)–NOR (2)

### 2.3. Problem description

For the CFDAMA scheme, the satellite on-board scheduler fills the requests into the resource assignment table (reservation request table and free assignment table) according to the FIFO (first in first out) principle, and then the scheduler will provide service from the top of the reservation request table by demand assigning contiguous slots to the corresponding user based on the number of slots requested. In the free assignment stage, it is achieved by assigning successive slots, one by one, to the user currently at the head of the free assignment table, moving each user to the bottom of the table after each slot assignment. As you can imagine that with the increasing amount of burst data traffic, the request slots in a frame can not be assigned to all users, so the users who need more slots may be placed at the back of the resource assignment table, thus the slots assignment can not be obtained, which will also cause the accumulation of data traffic and the increase of mean end-to-end delay. In the actual network, the types of service are different, which include real-time service such as voice and non-real-time services such as data. Different type of service has different requirements for network quality of service (QoS). If the user’s slot requests are prioritized, the priority of real-time services is higher than that of non-real-time services, and the priority of service with a large amount of data is higher than that of service with a small amount of data. The higher the priority of the user, the smaller the location of the reservation request table, which ensures that users with a large demand for real-time service and slots can obtain faster slots assignment and higher slots assignment right.

As mentioned above, there is a time delay T between the uplink and downlink frames, which represents the satellite onboard processing time. During this period of time, the user is still in a continuous burst data traffic process. However, the existing protocols do not process the burst data traffic during this period. With the increase of channel load, data packets will accumulate in the queue, which will lead to the decline of mean end-to-end delay performance. By the prediction strategy, if the current user is in a continuous burst state, we can predict the amount of data packets that will arrive in the time delay. The total number of slots requested by the user is the sum of the current number of slots and the number of slots obtained by the prediction. This prediction strategy can effectively reduce the accumulation of data packets in the queue.

## 3. A CLSTM and transfer learning based CFDAMA scheme

In recent years, machine learning also shows better performance in data prediction [20]. Compared to traditional learning methods, the prediction accuracy of LSTM is greatly improved. Because of the ability of extracting the hidden features between data by the convolutional neural networks (CNN), it can be used to improve the processing speed, so the convolutional long short-term memory networks (CLSTM) is an effective method in data prediction. The practice has shown that transfer learning performs very well when the distribution of data used in training and testing are different from each other. However, the prediction efficiency of the CLSTM network becomes worse when the period time of the data traffic increases. So, in this paper, we propose a new prediction strategy that combines CLSTM with transfer learning to improve the prediction performance.

### 3.1. Principle of CLSTM

#### 1. LSTM

Long short-term memory (LSTM), proposed in 1997 by Sepp Hochreiter et al [21,22], is a deep learning method. LSTM is an improved model of the traditional neural network model, which has a number of layers of nonlinear transformation.

**Fig 2:**
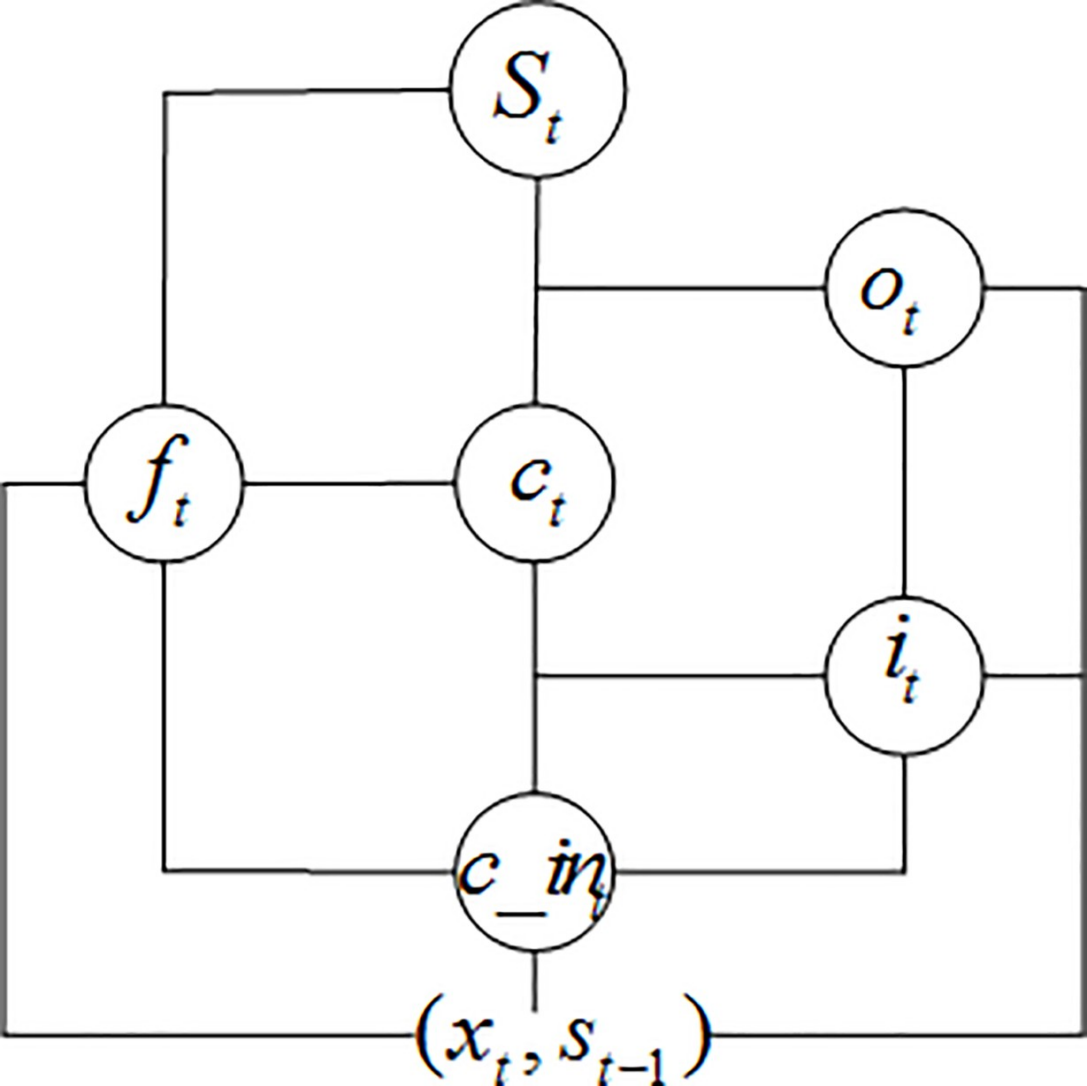
The basic structure of LSTM.

LSTM network always consists of three network layers, an input layer, an output layer, and some the hidden layers that accordance with different situations [23]. The hidden layer, which is shown in Fig 2, is composed of the memory cell. By this figure, we can see that one cell consists of three gates (input gate, forget gate and output gate) and a recurrent connection unit. Here, the input to this unit is *x*_*t*_. *s*_*t*_ is the current hidden state. Then, the current output is *o*_*t*_, and *c*_*t*_ is the internal memory of the unit. Gates use a sigmoid activation (denoted by *g*), while input and cell state are often transformed with tanh. LSTM cell can be defined as formulas (3)–(8) [24].

Input gate:

$$i_{t}=g(W_{xi}x_{t}+W_{hi}s_{t-1}+b_{i})$$ (3)

Forget gate:

$$f_{t}=g(W_{xf}x_{t}+W_{hf}s_{t-1}+b_{f})$$ (4)

Output gate:

$$o_{t}=g(W_{xo}x_{t}+W_{ho}s_{t-1}+b_{o})$$ (5)

Input transform:

$$c\_in_{t}=tanh(W_{xc}x_{t}+W_{hc}s_{t-1}+b_{c\_in})$$ (6)

State update:

$$c_{t}=f_{t}.c_{t-1}+i_{t}.c\_in_{t}$$ (7)

$$s_{t}=o_{t}.tanh(c_{t})$$ (8)

where *W*_*ij*_ is the connection weights of neuron *i* to *j*, *b* is deflection.

**Fig 3:**
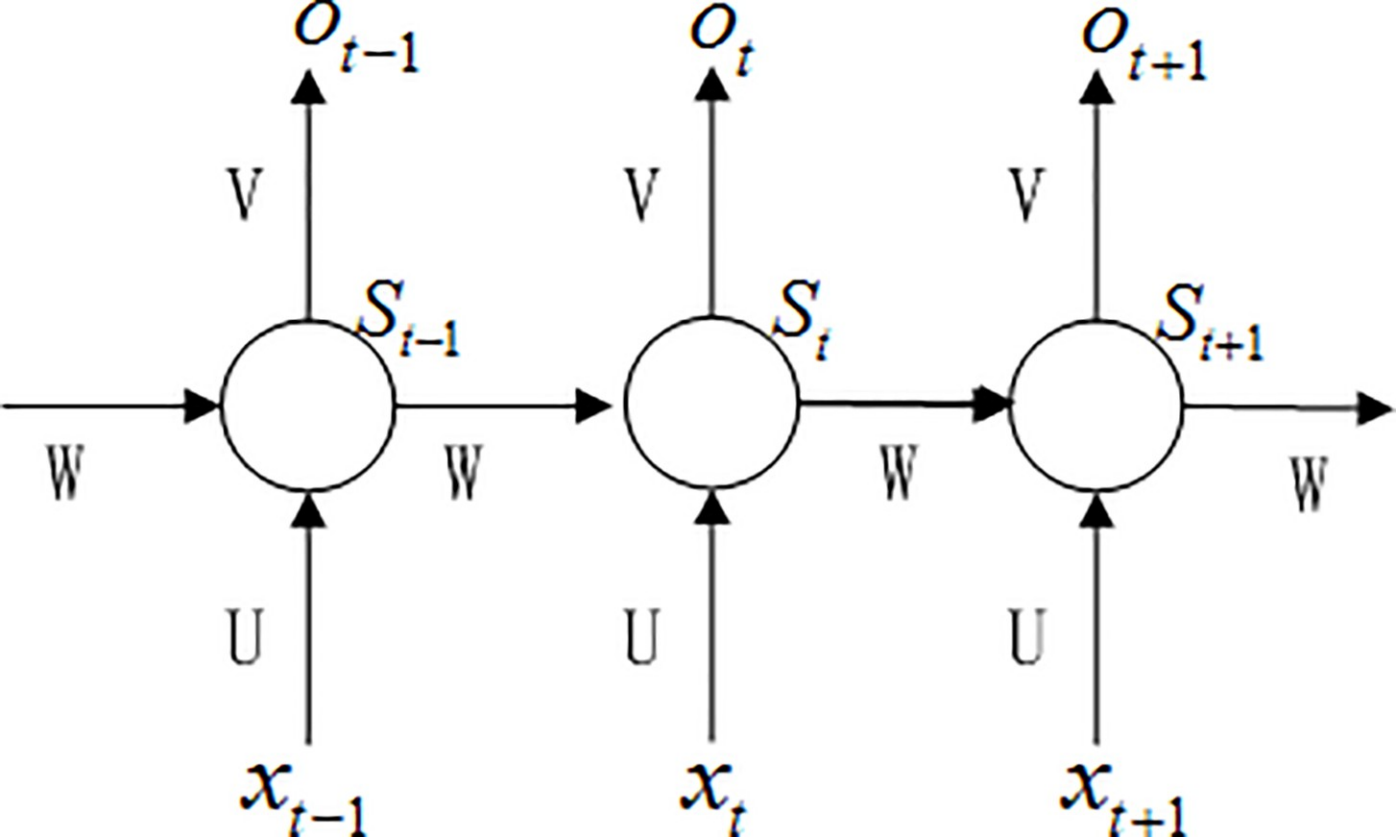
Network structure unfolded in time.

Fig 3 shows the network structure of LSTM, where *U*,*V*,*W* are the network parameters.

#### 2. CLSTM

**Fig 4:**
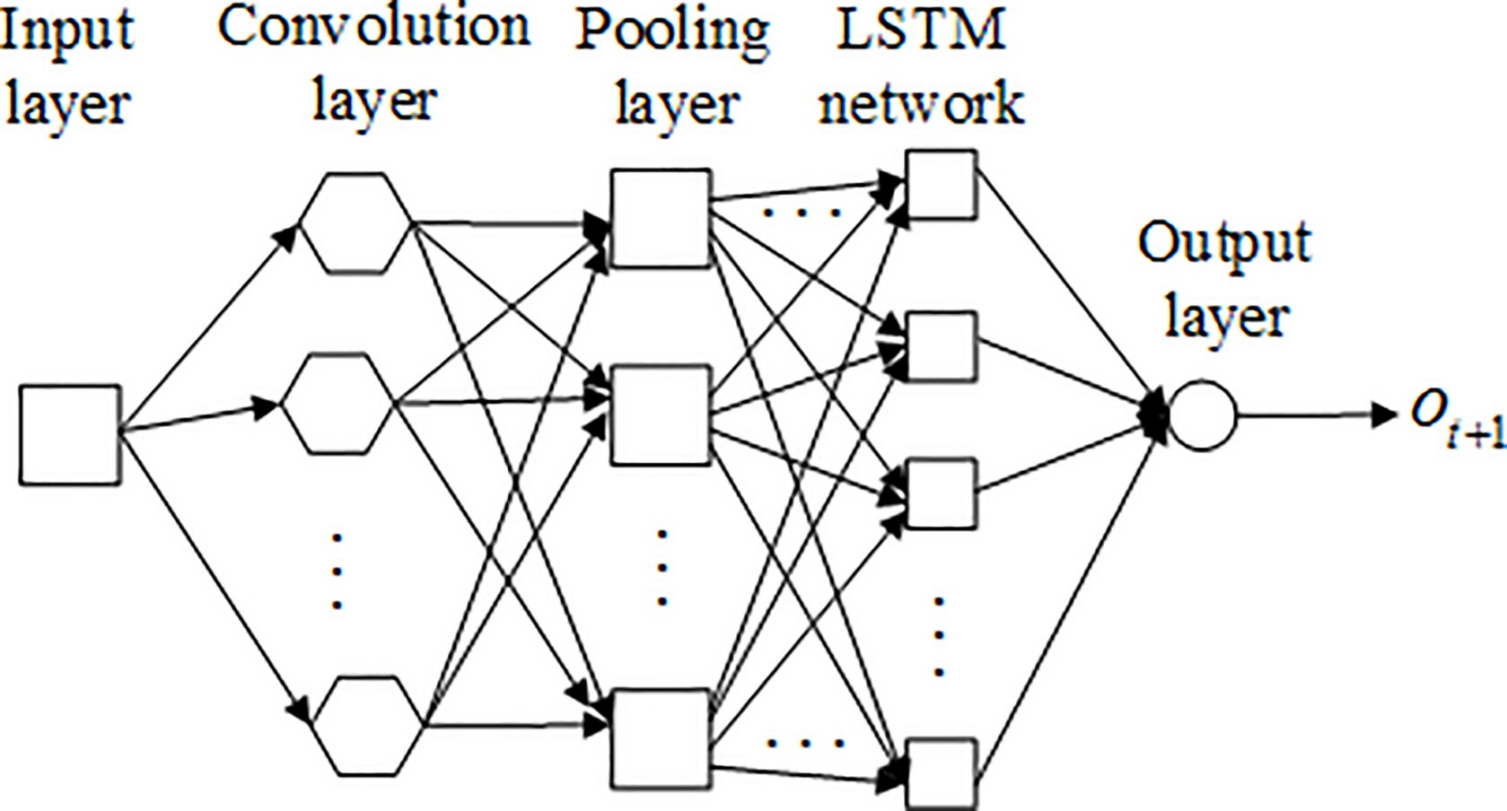
Network structure of CLSTM.

The principle of CLSTM is to cascade the convolutional neural network and LSTM [25], where the convolutional neural network acts as a filter, and it can extract the hidden features of the input data. The network structure of the CLSTM is shown in Fig 4. With this figure, we know that the biggest change of CLSTM is that there are a convolution layer and a pooling layer between the input layer and the LSTM network. The function of the convolution layer and pooling layer can be described as:

1. The convolution layer can help to realize local sense, which can be used to get a global sense.
2. The pooling layer can be used to lower the dimension of data and it can compress data.

The workflow of CLSTM can be described as:

1. Input the data, and preprocess the data.
2. Extract the characteristics of the data and compress the data by the convolution layer and pooling layer.
3. Take the output of the previous step as the input of the LSTM network, and then adjust the number of hidden layers and the length of time series of the LSTM network to get the ideal output.

CLSTM is a new scheme that can not only extract the features of the input data but also can filter the useless data to leave useful data. With these advantages, the amount of data processed by LSTM will be reduced and the processing speed of LSTM will be improved.

### 3.2. CLSTM based data traffic prediction strategy

In recent years, the statistical study of a large number of high-speed network traffic grouping data shows that the network data flow has obvious self-similarity characteristics, so the time series prediction model can better predict the actual traffic in the network.

**Fig 5:**
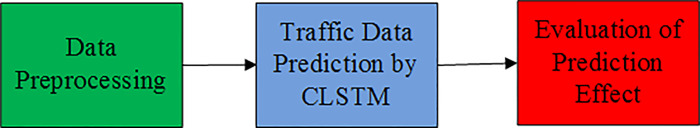
Data prediction based on CLSTM.

In the prediction model, the historical data are always selected as samples. According to Fig 3, the prediction model can be denoted by formula (9)

o(t+1)=F(o(t),o(t−1),…,o(t−n)) (9)

where *o*(*t*+1) is the predicted traffic data of a new time, *o*(*t*),*o*(*t*−1),…,*o*(*t*−*n*) are the current and past observed traffic data. The prediction model and network structure of CLSTM is shown in Fig 4. In this model, the predicted data is the output. Then, Fig 5 shows the flow of data prediction based on CLSTM.

#### 1. Data preprocessing

The traditional processing methods had met the bottleneck to deal with these large data sets. So, the input data are usually standardized. The purpose of data preprocessing is to convert the data into dimensionless pure values. After that, all these data that with different unit or scale of the indicator can be compared or weighted.

The data standardization method used in this paper is Z-SCORE [26], which standardizes the mean and standard deviation of the original data. After the data standardization, it meets the standard normal distribution. That is to say, the mean value of these data is 0, and the standard deviation of these data is 1. It is generally defined as follows:

$$Y(x)=\frac{x-\bar{x}}{\sigma }$$ (10)

where $\bar{x}$ is the average of all elements, *σ* is the standard deviation of all elements.

#### 2. Data Prediction by CLSTM

The process of data prediction done by the CLSTM scheme is described as follows.

Input: *x*_*t*−*n*_,*x*_*t−n*+1_,…,*x*_*t−k*_,*x*_*t*−*k*+1_, the observed historical data traffic.

1. Determine the number of layers of the CLSTM network according to the specific problem.
2. Determine the abandonment rate of each network node. In order to prevent over-fitting, the default value of this paper is set to 0.5.
3. Determine the iteration updating method of weight parameters.
4. Determine the epoch and batch size of model training.
5. Train the CLSTM network by the parameters determined by the above steps and the input data.
6. Predict the data traffic of *x*_*t*−*k*+2_.

#### 3. Evaluation method

In order to verify the prediction performance of the model, three evaluation parameters, RMSE (Root Mean Error), MAPE (Mean Absolute Percentage Error), and MAE (Mean Absolute Error) are used in this paper. These evaluation methods are given as (11)–(13) [27]. Where *N* is the number of samples, *y*_*t*_ is the actual value of time *t*, and ${\hat{y}}_{t}$ is the predicted value.

$$MAPE=\frac{1}{N}\sum_{t=1}^{N}\left|\frac{y_{t}-{\hat{y}}_{t}}{y_{t}}\right|\times 100\%$$ (11)

$$MAE=\frac{1}{N}\sum_{t=1}^{N}\left|y_{t}-{\hat{y}}_{t}\right|$$ (12)

$$RMSE=\sqrt{\frac{1}{N}\sum_{t=1}^{N}{\left(y_{t}-{\hat{y}}_{t}\right)}^{2}}$$ (13)

#### 4. Overview of the CLSTM algorithm

The pseudo-code for data traffic prediction by CLSTM is given in algorithm 1.

**Algorithm 1: Data Prediction by CLSTM**

Input: *x*_*t*−*n*_,*x*_*t−n*+1_,…,*x*_*t−k*_,*x*_*t*−*k*+1_.

Output: *x*_*t*−*k*+2_, a data predicted by CLSTM.

1. Data preprocessing according to 1;

2. Establishment of data traffic prediction model by LSTM according to the steps 1)- 6) in data traffic prediction by CLSTM;

3. Predict the traffic data of *x*_*t*−*k*+2_;

4. Evaluate the effect of prediction by formulas (11)–(13);

5. Output the traffic data of *x*_*t*−*k*+2_;

### 3.3. Principle of transfer learning

Transfer learning [28] is a new machine learning method that uses existing knowledge to solve problems in different but related fields. It relaxes two basic assumptions in traditional machine learning, aiming at transferring existing knowledge to solve the problem of learning with or without labels in the target domain. The following is a brief introduction to transfer learning.

A source domain, which is represented by *D* = {*x*,*P*(*x*)}. It can be divided into two parts:

1. Feature space *x*;
2. Marginal distribution probability *P*(*x*);

A learning task, which is represented by *T* = {y,*f*}. It can be divided into two parts too:

1. Label space *y*;
2. An objective prediction function *f*, which establishes the prediction of {*x*,*y*}.

Given source domain D_*S*_ and learning task *T*_*S*_, target domain *D*_*t*_ and learning task *T*_*t*_. The goal of transfer learning is to use the knowledge inD_*S*_ and *T*_*S*_, to improve the learning of target prediction function f in *D*_*t*_. D_*S*_≠*D*_*t*_, or *T*_*S*_≠*T*_*t*_.

**Fig 6:**
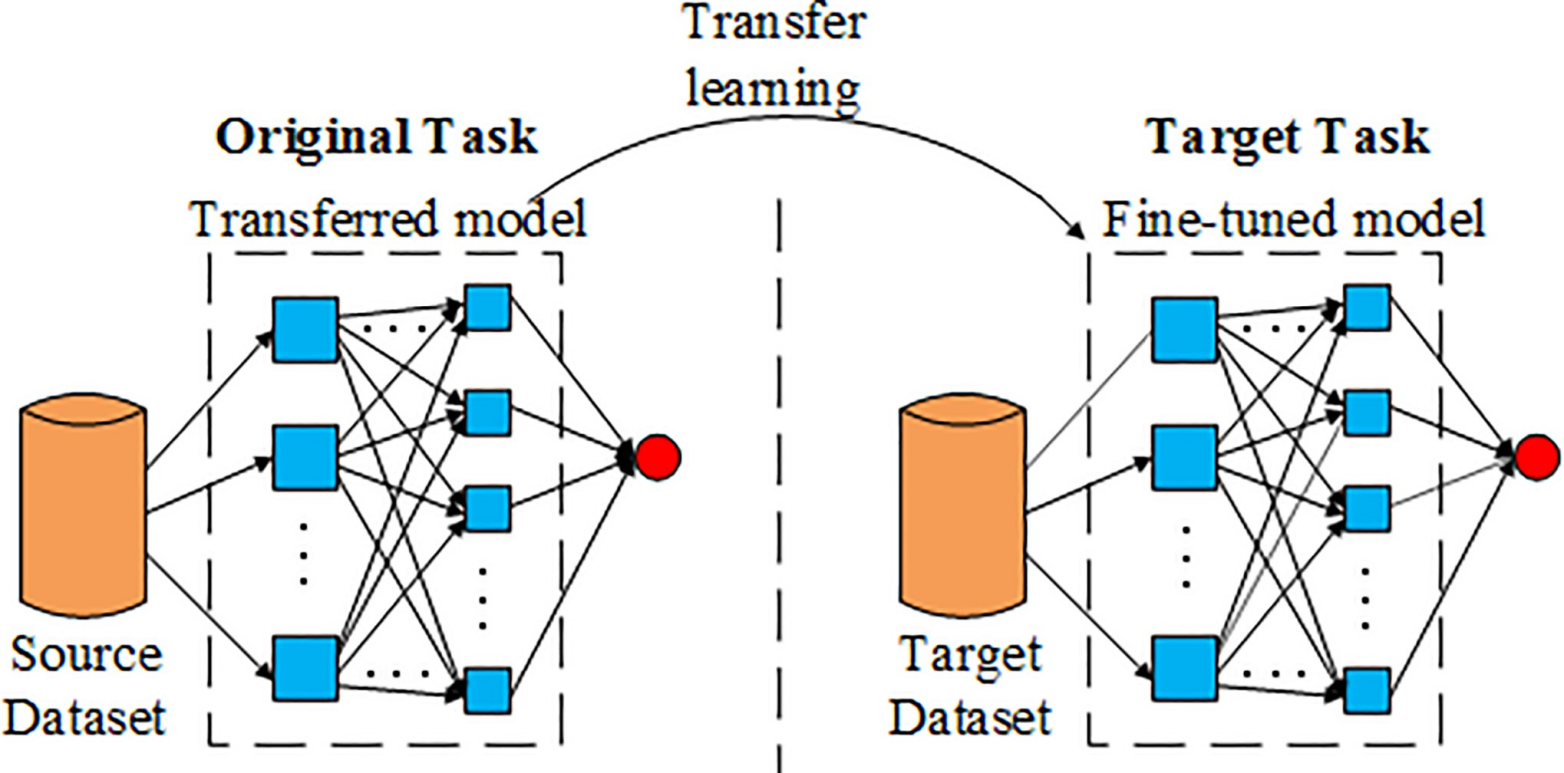
Principle of transfer learning.

When the source domain has a large amount of calibration data and the target domain has only a small amount of calibration data, and the source domain and the target domain have some common generalization characteristics, the transfer learning can be used to apply the data pre-training model of the source domain to the task of the target domain to improve the performance of the model in the target domain [29]. In this paper, the time series data transfer learning refers to train the CLSTM network by the time series data, and then transfer the characteristic of the trained CLSTM network to a new CLSTM network and try to fine-tune the parameters of the new network by a new time series data. Finally, the prediction of future data traffic is get. The principle of transfer learning in this paper is shown in Fig 6.

### 3.4. CLSTM and transfer learning based CFDAMA strategy

#### 1. CLSTM and transfer learning based data prediction scheme

In this paper, a new strategy of data prediction based on CLSTM and transfer learning (CLSTMTL) is proposed. The purpose is to predict the traffic data that generated during the period of T described above, so as to reduce the accumulation of data packets. This strategy can be described as follows: firstly, we can divide the input data into several data sequences of a certain length *l*(*l*≤*n*) and sort them in time order. Secondly, we will train the CLSTM network by the data of the first data sequence. Thirdly, transfer the characteristic parameters of the network to a new network and try to fine-tune the characteristic parameters of the new network. Fourthly, recycle and fine-tune the new network until all data sequences are trained. Finally, we can predict the data traffic by the trained network.

The pseudo-code of CLSTMTL is given in algorithm 2.

**Algorithm 2: CLSTMTL**

Input: *x*_*t*−*n*_,*x*_*t*−*n*+1_,…,*x*_*t*−1_,*x*_*t*_, the data that observed.

Output: *x*_*t*+1_, a data predicted by CLSTMTL.

1. Data preprocessing according to formula (10);

2. Divide the input data into *N* parts with the length of *l*(*l*≤*n*); /*Remark 1*/

3. Set up the initial parameters, *K* = 1;

4. for(*K* = 1;*K*≤*N*;*K*++)

{

5. Train CLSTM by the data traffic of *K*-th part; /*Remark 2*/

6. Transfer learning; /*Remark 3*/

}

    end

7. Predict the data traffic of *x*_*t*+1_;

8. Evaluate the effect of prediction according to (11)–(13);

9. Output the data *x*_*t*+1_;

Remark 1: The first part of data traffic is *x*_*t*−*n*_,*x*_*t*−*n*+1_,…,*x*_*t*−*n*+*l*−2_,*x*_*t*−*n*+*l*−1_, the second part is *x*_*t*−*n*+*l*_,*x*_*t*−*n*+*l*+1_,…,*x*_*t*−*n*+2*l*−2_,*x*_*t*−*n*+2*l*−1_, and then the *N*-th part is *x*_*t*−*n*+(N−1)*l*_,*x*_*t*−*n*+(N−1)*l*+1_,…,*x*_*t*−1_,*x*_*t*_.

Remark 2: If *K* = 1, then the first part of data is *x*_*t*−*n*_,*x*_*t*−*n*+1_,…,*x*_*t*−*n*−*l*−2_,*x*_*t*−*n*−*l*−1_, and then train the LSTM network by these data.

Remark 3: Transfer the characteristic parameters of the trained CLSTM network to a new CLSTM network and try to fine-tune the parameters of the new network by (*K*+1)-th data traffic.

**Fig 7:**
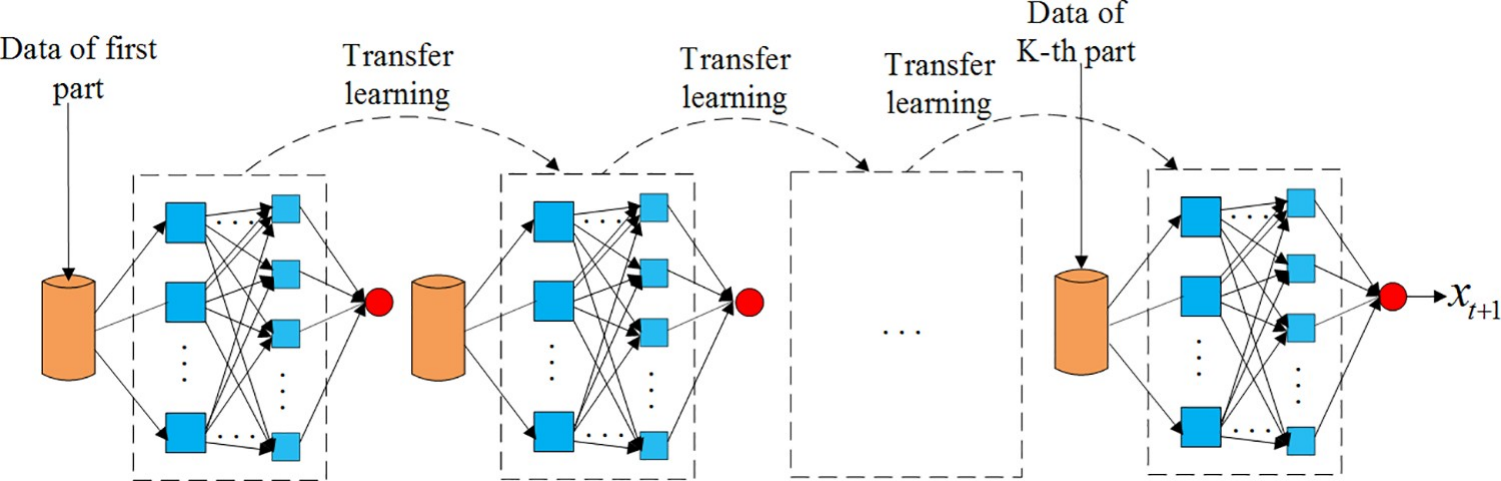
Principle of CLSTMTL.

In order to promote the prediction performance, the CLSTMTL scheme trains the CLSTM network firstly, and then transfers the network parameters to a new CLSTM network, and adjusts the parameters of the new network. After iterating many times, there will be a better prediction performance. The principle of the CLSTMTL scheme is shown in Fig 7.

#### 2. Slots requested by a user

When a user needs to access the communication networks, the slots requested by the user is the sum of the slots needed by the data in the queue and the slots needed by the data predicted by the previous step.

#### 3. Resource scheduling strategy of the satellite

This paper proposes a new resource scheduling strategy, which can achieve priority ranking. This strategy is that the satellite prioritizes the users according to the number of slots requested by the users, and then sets the priority number. The higher the priority of the user who requests more slots, the higher the position in the on-demand allocation table, which ensures that the user who needs more slots can obtain faster slots allocation and higher slots assignment rights, and achieve better mean end-to-end delay performance under high burst data traffic.

#### 4. CFDAMA-CLSTMTL scheme

Then we propose a new MAC protocol that introduce the CLSTMTL scheme into the CFDAMA protocol, and it is entitled CFDAMA-CLSTMTL. The pseudo-code of CFDAMA-CLSTMTL is given in algorithm 3.

**Algorithm 3: CFDAMA-CLSTMTL**

1. The user predict the data generated in the period of delay time;

2. The user calculate the number of slots;

3. The user send the slots request to the satellite scheduler;

4. The satellite scheduler allocates slots to the users;

Here, when the user calculates the number of slots in step 2 of algorithm 3, the number of slots is the sum of the slots needed by the data in the queue and the slots needed by the data predicted by CLSTMTL. In addition, the satellite allocates slots to the users according to the resource scheduling strategy of the satellite.

## 4. Simulation study

This section provides representative simulation results to verify the effectiveness of the proposed CLSTM and transfer learning based CFDAMA scheme. In this section, the CFDAMA-CLSTMTL scheme has been simulated by the combination of MATLAB, STK, and OPNET. The MATLAB is used to generate data traffic, and STK is used to generate satellite orbital data, and then we import the orbital data and data traffic into OPNET to get the simulation results.

### 4.1. Data traffic model

**Fig 8:**
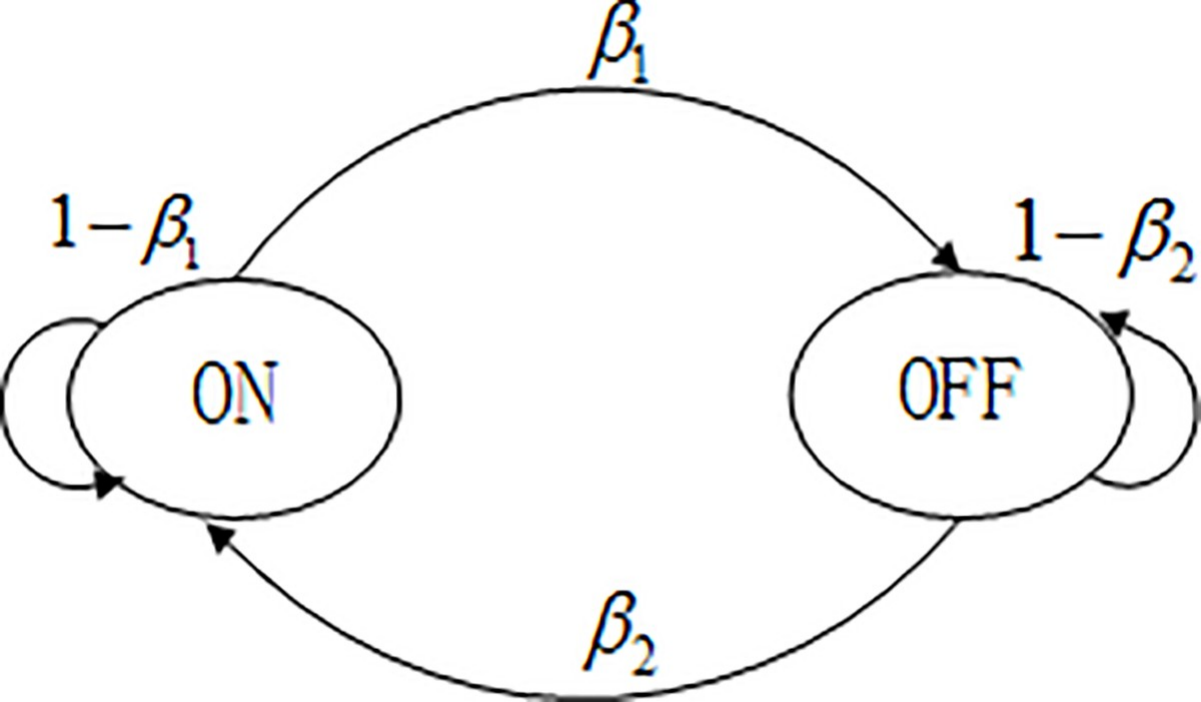
ON-OFF traffic model.

A large number of experimental studies have proved that a correct traffic model is important to evaluate the performance of the MAC protocol. In this paper, a Pareto ON-OFF traffic model [30] is used in the simulation. The state transition diagram of the ON-OFF model is shown in Fig 8. The traffic model consists of several independents ON and OFF traffic durations. The traffic data is generated when it is ON, and the transfer probability is *β*_1_. There is no data when it is OFF, and the transfer probability is *β*_2_. The number of data packets is determined by a given distribution function, and the interval of the arrival of data packets also obeys a certain distribution.

**Fig 9:**
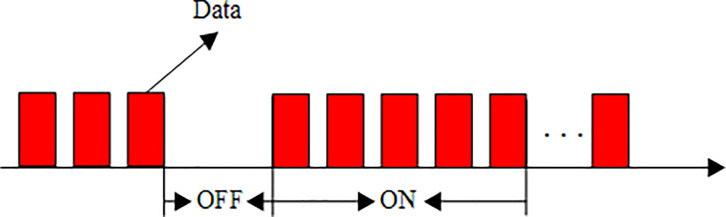
Pareto ON-OFF traffic data model.

The traffic data in modern high-speed networks has obvious self-similarity. The self-similar traffic means that the data is bursty over a wide range of time scalavy tailes. The Pareto distribution can be described as formula (14), in which *λ*_1_,*λ*_2_,*ε*_1_,*ε*_2_ are the parameters of time scalavy tailes and minimum duration of ON and OFF respectively. The data traffic model is shown in Fig 9.

$$\begin{array}{l} f_{ON}(t_{ON})=\lambda_{1}\varepsilon_{1}^{{}^{\lambda_{1}}}t_{ON}^{{}^{-\lambda_{1}-1}},\lambda_{1},\varepsilon_{1}\geq 0,t_{ON}\geq \varepsilon_{1}\\ f_{OFF}(t_{OFF})=\lambda_{2}\varepsilon_{2}^{{}^{\lambda_{2}}}t_{OFF}^{{}^{-\lambda_{2}-1}},\lambda_{2},\varepsilon_{2}\geq 0,t_{OFF}\geq \varepsilon_{2} \end{array}$$ (14)

### 4.2. Simulation scenario

**Fig 10:**
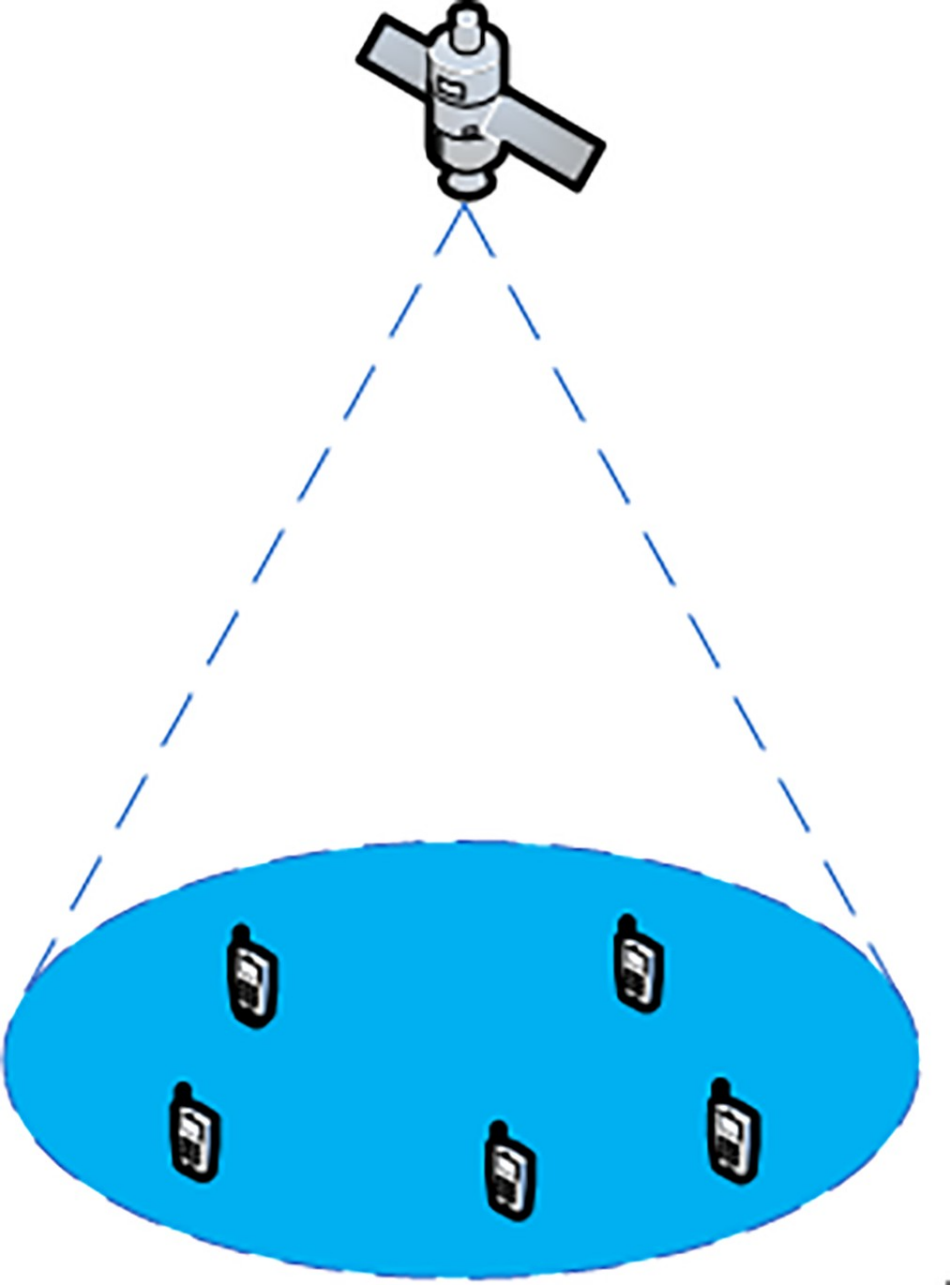
Simulation scenario.

The network model of the simulation for CFDAMA-CLSTMTL is composed of Inmarsat synchronous satellites and many ground nodes. The simplified system model of the simulation scenario is shown in Fig 10. As illustrated in Fig 10, consider the satellite communication system in which the satellite serves the ground users in its coverage. The Inmarsat satellite has an onboard scheduler, which can forward frames. The ground nodes consist of several users, which access satellite network by the CFDAMA-CLSTMTL protocol. The simulation parameters for the satellite networks, which can help to establish the satellite communication networks, are given in Table 4. Then, the simulation parameters for the CLSTM network, which can help to establish the CLSTM network, are given in Table 5.

**Table 4:** Simulation parameters for the satellite networks.

Parameters	Setting
Number of satellite	13
Altitude of satellite (km)	35700
Number of ground stations	5,10,20,40,80
channel rate (Mbit/s)	2
Number of Carriers per Frame	8
Number of slots per carrier	128
Number of bits per slot (bit)	424
Time length per frame (s)	0.0027136
Channel Load	0.1–1.0
Source Model	Poisson
	Pareto ON-OFF
Minimum duration of ON and OF	*ε*_1_ = *ε*_2_ = 1.0
Parameters of time scalavy tailes	*λ*_1_ = *λ*_2_ = 1.2

**Table 5:** Simulation parameters for the CLSTM network.

Parameters	Setting
Number of input nodes	8
Number of output nodes	24
Time step	72
Number of Hidden Nodes	1000
learning rate	initial value 7e-6
Activation function	ReLU
Batch size	24
Number of iterations	1000

### 4.3. Experimental results and analysis

This paper examines the comparative performance of the CFDAMA-CLSTMTL scheme with CFDAMA-PA and CFDAMA-PB, and the experimental results and analysis are given below. One thing we need to emphasize is that in all our experiments, the parameters are set the same. In other words, for these three different schemes, we have used the same parameters and we do not tune the parameters in all these experiments.

**Fig 11:**
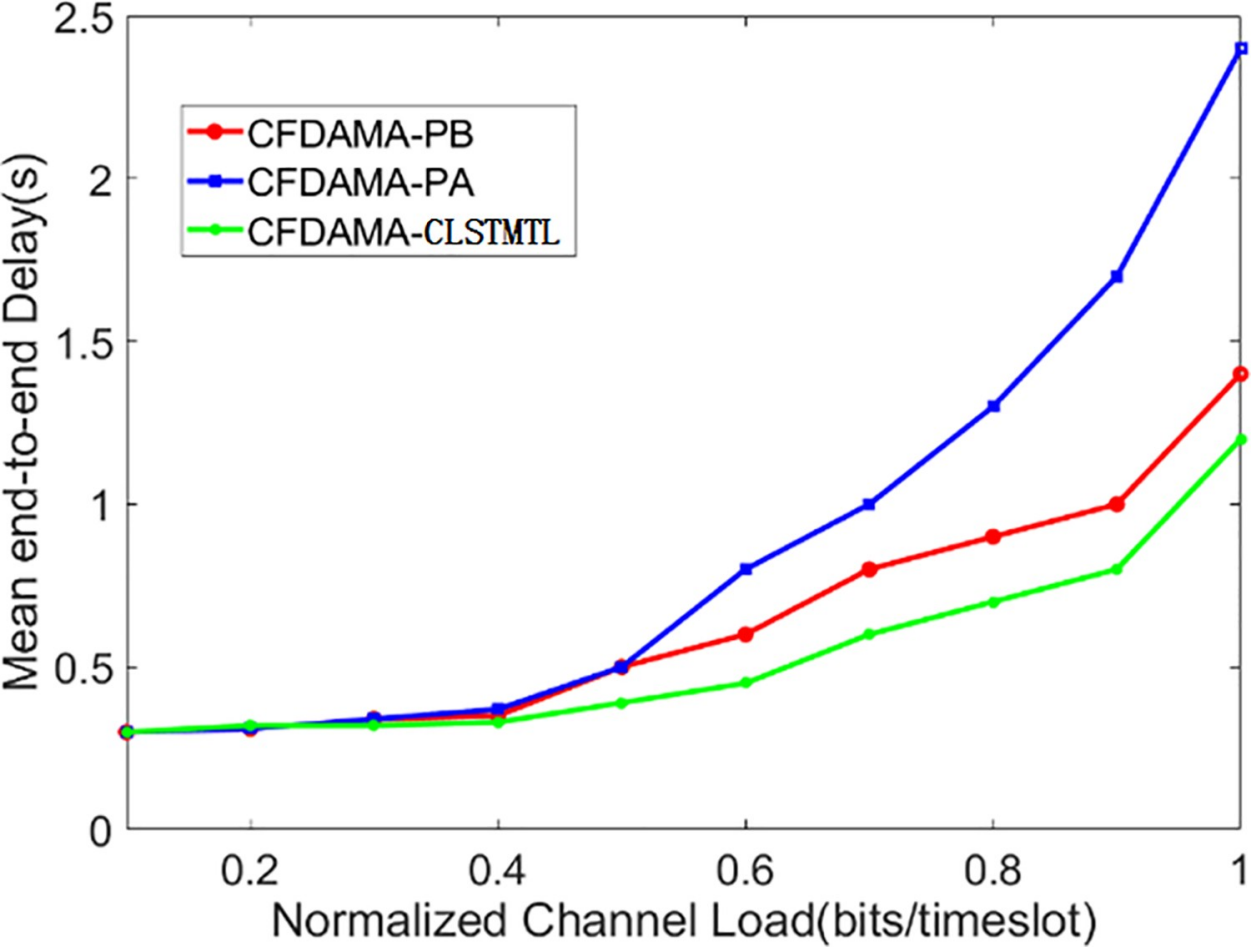
Mean end-to-end delay/normalized channel load performance.

Firstly, the results of the condition of a fixed number of users but varying normalized channel load are given to evaluate the mean end-to-end delay performance of these three schemes. Fig 11 shows the results of the mean end-to-end delay/normalized channel load, where the number of users is 40 and the channel load is between 0.1 and 1.0.

As can be seen from Fig 11, when the normalized channel load is 0.1–0.4, the three schemes show similar mean end-to-end delay/normalized channel load performance, but the performance of the CFDAMA-PA scheme increases sharply when the channel load is 0.5–1.0. The reason is that with the increases of normalized channel load, the burst data traffic increases, and the number of user’s requests in the system will increase too. If the number of slots assigned on-demand at this time exceeds the total available slots of a frame, the users with a large demand for slots behind the on-demand assignment table will not get enough slots, which will increase the mean end-to-end delay. The CFDAMA-PB scheme increases the flexibility of slots assignment by piggybacking requests, which can avoid setting fixed slots to store user’s requests at high normalized channel loads. However, if there are too many users, it will also lead to users with large slot requirements can not be assigned enough slots. The CFDAMA-CLSTMTL scheme assigns slots to users with slot requests when normalized channel load increases, and guarantees priority for data packets of users with large slots demand. Therefore, the mean end-to-end delay/normalized channel load performance is better than that of CFDAMA-PA and CFDAMA-PB.

**Fig 12:**
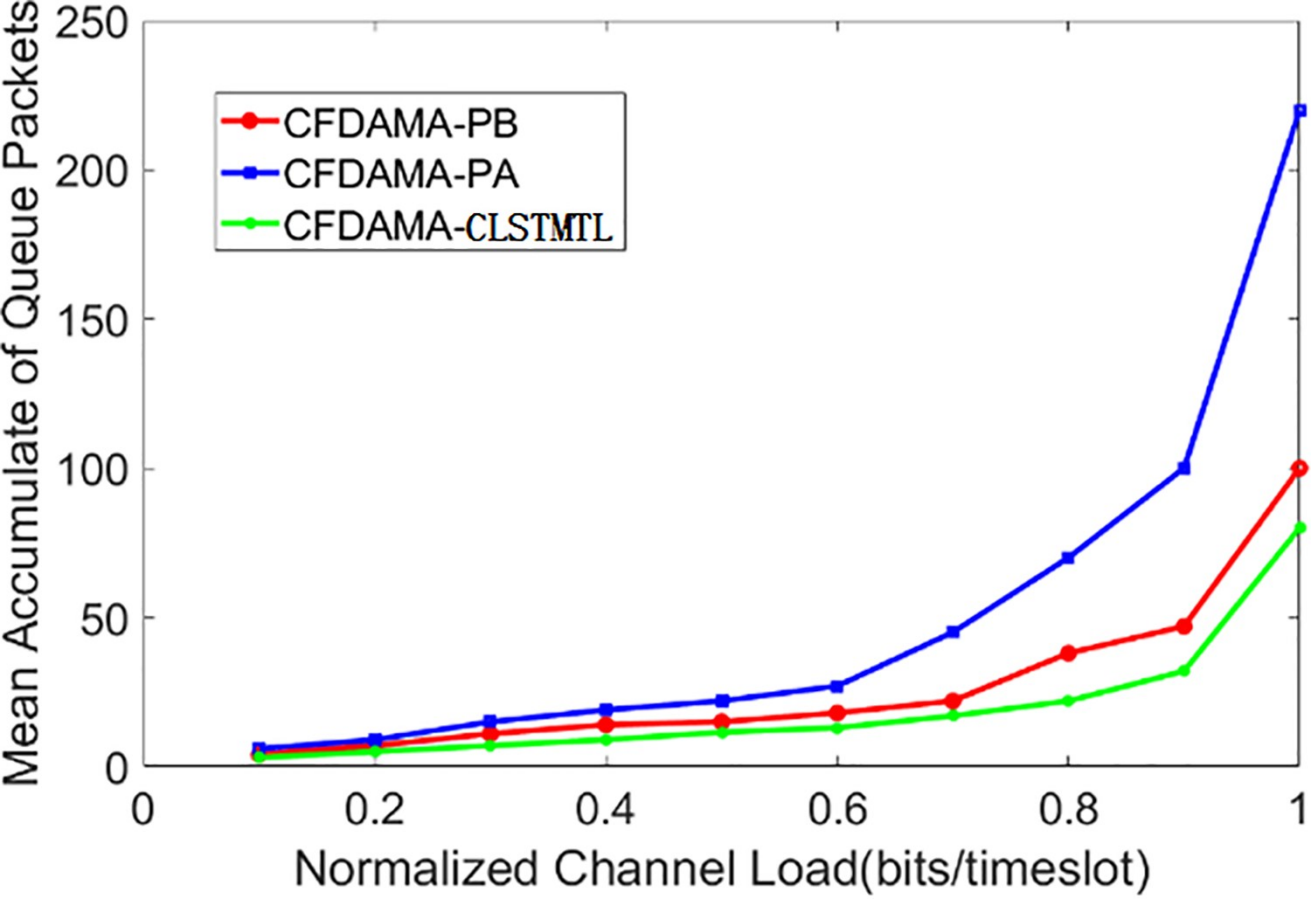
Mean accumulate of queue packets/normalized channel load performance.

Then, Fig 12 shows the results of the mean accumulate of queue packets/normalized channel load. As can be seen from it, the cumulative number of data for the three schemes is not much different in the range of normalized channel load 0.1–0.4, which is because the availability of system resources is high when the channel load is small, and users can send the data in the queue in time. From the normalized channel load of 0.5, the performance of CFDAMA-PB and CFDAMA-CLSTMTL is obviously superior to CFDAMA-PA, and CFDAMA-CLSTMTL presents the best performance, which is because CFDAMA-PA has fixed slot points in the frame. It is likely that there are some users do not has requested when the next frame arrives, while a larger part of users have requests, but can not get the corresponding slots to send it. The CFDAMA-PB scheme does not take certain measures to the burst data traffic, and can only calculate the data in the current queue in the case of high burst, which will result in the continuous accumulation of packets. In contrast, the CFDAMA-CLSTMTL scheme predicts the data traffic, and if there is a burst of data coming at the next moment, the number of slot requests send at the current time is the sum of the number of slots for the existing data traffic and the number of slots for the predicted data traffic. For the CFDAMA-CLSTMTL scheme, the data accumulation will still increase when the channel load is very high, which is because the number of current data packets exceeds the capacity of the whole system.

**Fig 13:**
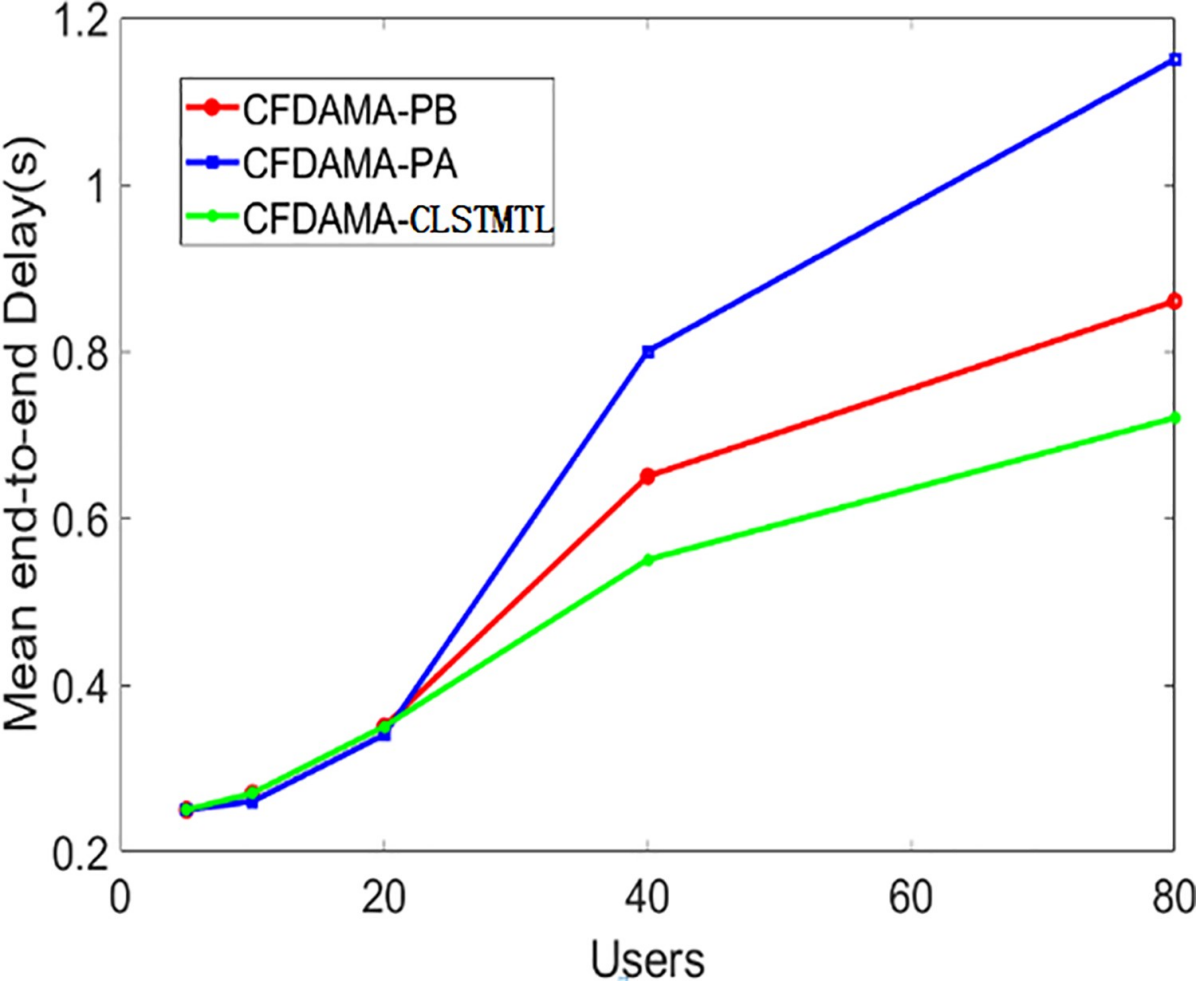
Mean end-to-end delay/number of users performance.

**Fig 14:**
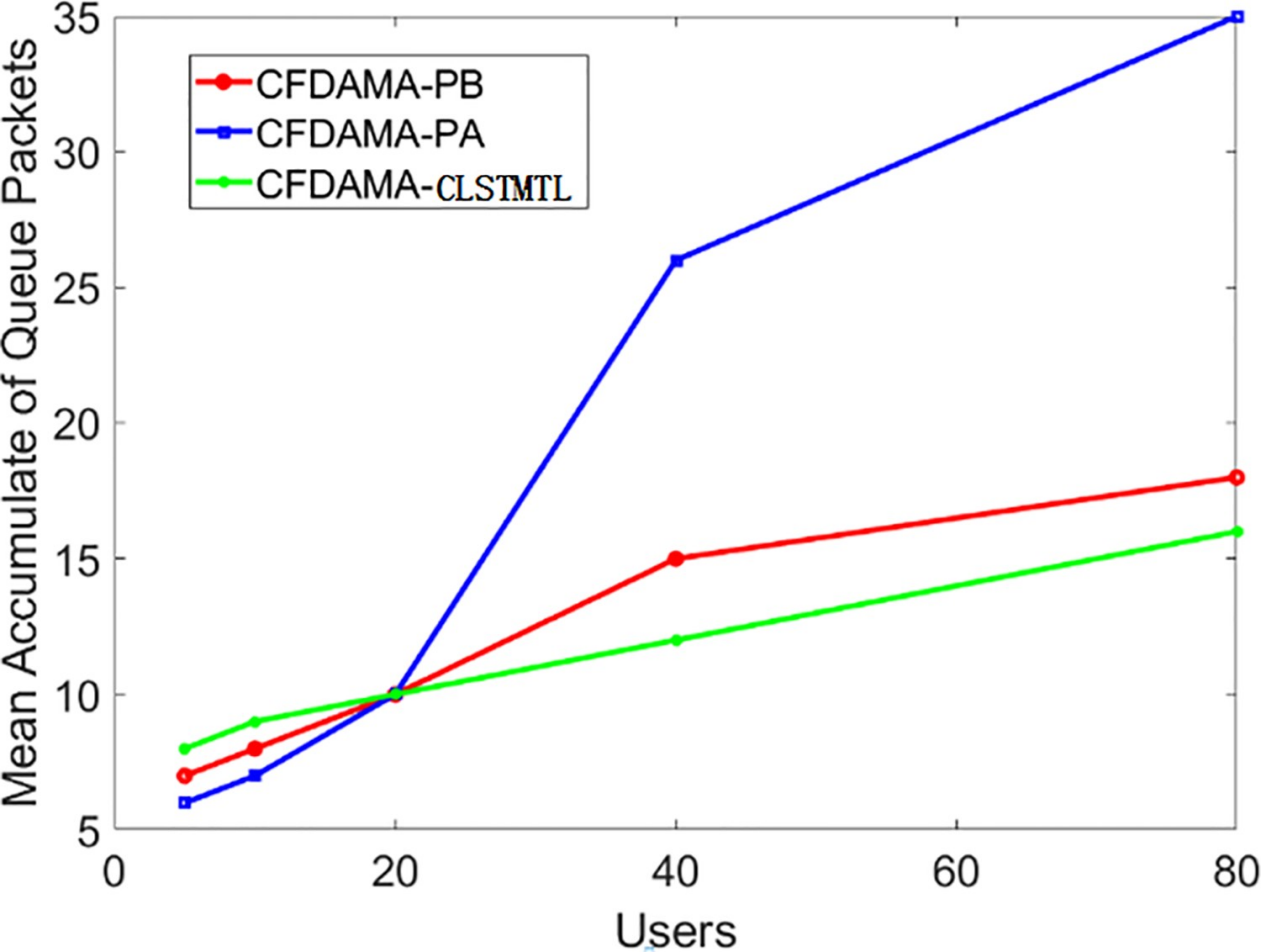
Mean accumulate of queue packets/number of users performance.

Then, there is another experiment with the conditions of fixed channel load but a variable number of users, which can get the performance of CFDAMA-CLSTMTL in access fairness. As shown in Figs 13 and 14, the channel load is 0.6, and the number of users is 5, 10, 20, 40, and 80 respectively.

Fig 13 shows the results of mean the end-to-end delay/number of users. It can be seen from it that the performance of CFDAMA-PA is similar to CFDAMA-PB in the range of 5–20 users, which is superior to CFDAMA-CLSTMTL. The reason is that when the number of users is small, CFDAMA-PA can provide timely access to the users by fixed appointment slots. The scheduling process of CFDAMA-PB is similar to CFDAMA-PA, and all of these schemes’ fairness of users is also maximized. The mean end-to-end delay of the CFDAMA-CLSTMTL scheme is slightly larger than that of the previous two schemes because of the data prediction process and computational sequencing.

Fig 14 shows the results of the mean accumulate of queue packets/number of users. It can be seen from it that the CFDAMA-PB and CFDAMA-CLSTMTL can guarantee the real-time and fairness of users’ access by means of piggyback with the increasing number of users, which reduce the probability that users can not access in time in sudden situations and also reduce the data accumulation in the queues. At the same time, CFDAMA-PB and CFDAMA-CLSTMTL can guarantee the real-time and fairness of users’ access. The CFDAMA-CLSTMTL scheme can also reduce the mean end-to-end delay and data accumulation.

Meanwhile, in order to evaluate the performance of the prediction strategy, we should concern with the evaluation method by formulas (11)–(13). Table 6 depicts the evaluation results of the prediction performance of the proposed strategy. We found that the prediction performance of the proposed strategy for the training set and test set have shown improvements, which shows a small deviation from the true value.

**Table 6:** Evaluation results of the prediction performance.

Entry	RMSE	MAE	MAPE (%)
Training Set	0.312	0.265	4.61
Test Set	0.298	0.195	3.47

In summary, the proposed LSTM and transfer learning-based CFDAMA strategy shows good performance, and it can gratify the multiple access requirements of the broadband satellite system.

## 5. Conclusion

In this paper, we propose a new CFDAMA scheme, which introduces the combination of CLSTM and transfer learning and it is entitled CFDAMA-CLSTMTL. The motivation of this new strategy is to predict the data traffic in the time of the satellite on-board processing, so as to promote the performance of multiple access in the satellite communication networks. Generally speaking, when the user needs to communicate with each other by the satellite networks, it will send slots requests to the satellite. However, there will be a delay time T between the user’s requests and the reply. The slots requested by the user do not include the data generated within that delay time. In this paper, we propose a prediction strategy based on the combination of CLSTM and transfer learning, which can predict the data in the delay time. Then the slots requested by the users will be the sum of the slots needed by the data in the queue and the slots needed by the data predicted by the CLSTMTL scheme. At last, we introduce the prediction strategy into the CFDAMA scheme and do some experiments to verify the performance of this new strategy. The experimental results show that the CFDAMA-CLSTMTL scheme performs better than CFDAMA-PA and CFDAMA-PB in both mean accumulate of queue packets/normalized channel load and mean end-to-end delay/number of users with the same network conditions.
